# Appraising the causal relationship between kidney function and hearing performance: a two-sample Mendelian randomization study

**DOI:** 10.1080/0886022X.2026.2663653

**Published:** 2026-05-14

**Authors:** Weiling Huang, Zhao Li, Yue Zhang, Hongsheng Tan, Jianrong Shi

**Affiliations:** a School of Traditional Chinese Medicine, Shanghai University of Traditional Chinese Medicine, Shanghai, China; b Institute of Clinical Medicine & School of Public Health, Shanghai Jiao Tong University School of Medicine, Shanghai, China; c Department of Bioinformatics and Biostatistics, Shanghai Jiao Tong University, Shanghai, China; d Shanghai Jiao Tong University College of Basic Medical Sciences, Shanghai, China

**Keywords:** Mendelian randomization, chronic kidney disease, sensorineural hearing loss, estimated glomerular filtration rate, genome-wide association study, causal inference

## Abstract

Epidemiological studies have reported an association between chronic kidney disease and hearing impairment, but the causal relationship remains uncertain. We conducted a bidirectional two-sample Mendelian randomization study to examine whether genetically predicted kidney function traits causally influence speech reception threshold, an objective measure of auditory performance. Genetic instruments for estimated glomerular filtration rate, chronic kidney disease, and blood urea nitrogen were obtained from large-scale genome-wide association studies conducted by the CKDGen Consortium, and summary statistics for speech reception threshold in the left and right ears were derived from the UK Biobank. The inverse variance weighted method was used as the primary analytical approach, complemented by MR-Egger, weighted median, and mode-based estimators. No robust causal associations were identified between genetically predicted kidney function traits and SRT in either ear. IVW estimates for BUN, CKD, and eGFR were non-significant (all *P* > 0.05). Although the IVW estimate for eGFR and left-ear SRT approached nominal significance and some secondary analyses showed nominal associations, these findings were inconsistent across methods and did not meet our criteria for robust causal inference. Reverse Mendelian randomization analyses also showed no significant associations. Sensitivity analyses showed no evidence of directional horizontal pleiotropy, and outlier correction did not materially change the results. This study provides no robust genetic evidence supporting a causal relationship between genetically predicted kidney function traits and hearing function, as measured by speech reception threshold, in either direction under the current MR framework. The observed epidemiological association may instead reflect shared systemic, developmental, or environmental mechanisms.

## 1. Introduction

Hearing loss (HL) is a highly prevalent sensory disorder and a major contributor to disability worldwide [1–3]. According to the World Health Organization, over 1.5 billion people currently experience of HL, a number projected to rise to 2.5 billion by 2050 [4]. Beyond its impact on communication and quality of life, HL has increasingly been associated with systemic physiological dysregulation [5,6]. Patients with HL are also at higher risk for cognitive impairment and neuropsychiatric disorders, further compromising their overall quality of life [7,8]. Speech reception threshold (SRT), which reflects the ability to perceive and recognize speech signals, has emerged as a functionally relevant auditory phenotype in large population-based studies and has also been incorporated into genetic and genomic analyses of hearing-related traits [9]. Investigations have further incorporated SRT as a quantitative phenotype in hearing-related genetic and genomic analyses, supporting its utility in evaluating auditory function within epidemiological and genetic frameworks [10]. In this context, SRT offers an opportunity to investigate hearing performance using continuous, objectively derived data in large-scale genome-wide association study (GWAS) resources.

Chronic kidney disease (CKD) is a major global public health burden that affects an estimated 850 million individuals, accounting for approximately 11% of the world’s population, and is associated with substantial morbidity, mortality, and healthcare costs [11,12]. Due to its progressive nature and systemic involvement, monitoring kidney function–related biomarkers are essential for understanding their impact on other organ systems and for early disease prevention [13]. In addition to renal and cardiovascular complications, CKD has been linked to abnormalities in multiple organ systems, including the auditory system [14,15]. Epidemiological studies have reported associations between reduced kidney function and hearing impairment, and several reviews have emphasized shared vascular, metabolic, inflammatory, and developmental mechanisms between the kidney and the inner ear [16]. However, most of this evidence is observational or cross-sectional in nature and therefore remains vulnerable to residual confounding and reverse causation [17]. In particular, factors such as aging, diabetes, hypertension, microvascular injury, and systemic inflammation may contribute to both kidney dysfunction and auditory decline, making it difficult to determine whether kidney dysfunction itself directly causes impaired hearing performance.

Developmental and physiological links between the kidney and the auditory system further complicate the interpretation of observational associations. The kidney and the inner ear share common structural and molecular features, including ciliary function, fluid transport mechanisms, and basement membrane components, and several genes involved in oto-renal syndromes play key roles in the development of both organs [18–20]. These observations support a biologically plausible connection between renal and auditory traits, but they do not necessarily establish a direct downstream causal pathway from impaired kidney function to later hearing dysfunction. Instead, they may reflect shared developmental architecture or pleiotropic biological processes affecting both organs in parallel.

Mendelian randomization (MR) uses genetic variants as instrumental variables to strengthen causal inference by reducing confounding and reverse causation under specific assumptions. To our knowledge, no previous MR study has specifically evaluated whether genetically predicted kidney function influences SRT. We therefore performed a two-sample MR analysis to investigate whether three kidney function-related traits – estimated glomerular filtration rate (eGFR), CKD, and blood urea nitrogen (BUN) – are causally associated with left- and right-ear SRT. We selected SRT because it is a functionally relevant, continuous phenotype available in large-scale GWAS resources; however, we also recognize that SRT may not capture all dimensions of hearing impairment, particularly high-frequency peripheral auditory dysfunction. Accordingly, this study was designed to test whether genetically predicted kidney function affects speech reception performance, rather than all hearing phenotypes more broadly.

## 2. Materials and methods

**Figure 1.**
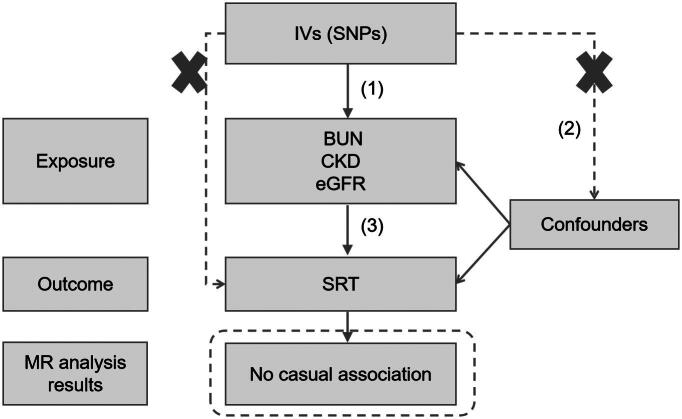
Study design.

We conducted a two-sample Mendelian randomization analysis to evaluate the potential causal effect of kidney function-related traits on auditory performance. In this framework, kidney function traits were treated as exposures and speech reception threshold (SRT) as the outcome. Three kidney-related traits were selected to represent complementary aspects of renal physiology and disease burden: estimated glomerular filtration rate (eGFR), chronic kidney disease (CKD), and blood urea nitrogen (BUN). eGFR reflects renal filtration capacity and is widely used for CKD staging and disease monitoring, whereas BUN provides additional information related to nitrogen metabolism and renal excretory function [21,22]. SRT was analyzed as the hearing-related outcome because it is a quantitative and functionally relevant phenotype available in large-scale GWAS datasets. A schematic overview of the study design is shown in Figure 1.

Genetic variants significantly associated with kidney function were selected as instrumental variables (IVs) [23,24]. To ensure the validity of the MR analysis, all instrumental variables were required to satisfy the three core MR assumptions: they were robustly associated with the exposure, independent of potential confounders, and influenced hearing ability only through their effects on kidney function.

### 2.1. Data sources for estimated glomerular filtration rate, chronic kidney disease, and blood urea nitrogen (exposure data)

Summary-level GWAS data for eGFR, CKD, and BUN were obtained from the CKDGen Consortium. For the present study, we used European-ancestry-specific summary statistics to reduce bias from population stratification. The eGFR GWAS included 765,348 participants after quality control, the CKD GWAS included 625,219 participants including 64,164 CKD cases, and the BUN GWAS included 416,178 participants. In the original contributing studies, eGFR was derived using established equations, CKD was defined as eGFR < 60 mL/min/1.73 m^2^, and BUN values were harmonized across studies where necessary [25,26]. Details of the contributing cohorts and phenotype derivation have been described previously by the original consortium [27].

### 2.2. Data source for hearing function (outcome data)

GWAS summary statistics for hearing function were obtained from the UK Biobank *via* the IEU OpenGWAS database. Hearing ability was assessed using speech reception threshold estimates, analyzed separately for the left ear (ukb-b-9052) and right ear (ukb-b-8316). Both traits were modeled as continuous variables and were derived from participants of European ancestry. The left-ear SRT GWAS included 146,131 individuals and the right-ear SRT GWAS included 146,031 individuals. All analyses were based on the HG19/GRCh37 genome build, with standardized genotype quality control and imputation procedures implemented in the original GWAS framework. Although the exposure data were obtained from CKDGen and the outcome data from UK Biobank/IEU OpenGWAS, complete exclusion of sample overlap between the datasets cannot be guaranteed.

### 2.3. Selection of genetic instruments (selection of genetic IVs)

Genetic variants significantly associated with each kidney function trait at the genome-wide significance threshold (*P* < 5e-08) were selected as candidate instrumental variables. To ensure independence between instruments, SNPs were linkage disequilibrium clumped using r^2^ < 0.001 within a 10,000 kb window. These SNPs were then harmonized with the corresponding SRT summary statistics. Instrument strength was assessed using the F statistic; in general, the F statistic for an individual SNP can be approximated as beta^2^/SE^2^, and all selected instruments exceeded the conventional threshold of 10, indicating low risk of weak instrument bias. We additionally applied MR-PRESSO [28] to detect potential outlier variants that might introduce horizontal pleiotropy. The final sets of instrumental SNPs are provided in the Supplementary Tables (listed in Supplementary Tables 1–3).

### 2.4. Mendelian randomization analyses

MR relies on three core instrumental variable assumptions: (1) the genetic variants are robustly associated with the exposure (relevance); (2) the genetic variants are independent of confounders of the exposure–outcome relationship (independence); and (3) the genetic variants influence the outcome exclusively through the exposure and not *via* alternative biological pathways (exclusion restriction). The primary causal estimates were obtained using the inverse variance-weighted (IVW) method under a multiplicative random-effects model [29]. Under the IVW framework, SNP-specific Wald ratios are combined to generate an overall estimate of the causal effect. Because the IVW approach can be sensitive to pleiotropy, we also performed several complementary MR methods, including MR-Egger [30], weighted median [31], simple mode, and weighted mode [32]. These approaches provide robustness under different assumptions regarding invalid instruments and horizontal pleiotropy.

### 2.5. Sensitivity analyses

To evaluate the robustness of the MR estimates, we performed a series of sensitivity analyses. Horizontal pleiotropy was assessed using the MR-Egger intercept test, and heterogeneity among SNP-specific estimates was assessed using Cochran’s Q statistic. MR-PRESSO was used to identify potential outlier variants and to compare raw and outlier-corrected estimates. Leave-one-out analyses were further conducted to determine whether any single SNP disproportionately influenced the overall effect estimate. Funnel plots and scatter plots were used to visualize heterogeneity and the distribution of SNP-specific effects across MR methods.

### 2.6. Statistics

All analyses were conducted using the TwoSampleMR (version 0.5.11) and MR-PRESSO (version 1.0) packages in R (version 4.4.0). For global-level tests, a two-sided *P*-value of 0.05 was considered statistically significant. Statistical power was calculated to estimate the minimum detectable effect size for continuous outcomes. The results are expressed as standardized *β* coefficients, representing the change in SRT per genetically predicted unit increase in exposure. In summary, results are considered significant if they meet the following stringent criteria: (1) the association using IVW reaches Bonferroni-corrected *P* < 0.05; (2) the association patterns are consistent across all MR methods; (3) the F-statistic for all IVs is > 10; (4) there is no significant heterogeneity among the IVs; (5) there is no evidence of horizontal pleiotropy (Egger intercept *P* > 0.05, and MR-PRESSO global test *P* > 0.05).

### 2.7. Ethics

This study utilized publicly available de-identified data from participant studies, which had received approval from the relevant ethics committees in accordance with standards for human experimentation. Therefore, no separate ethical approval was required for this research.

### 2.8. Bidirectional Mendelian randomization

To examine potential reverse causality, we conducted bidirectional MR analyses by reversing the exposure–outcome relationship. In this analysis, speech reception threshold (SRT) for the left and right ears was treated as the exposure, while kidney function–related traits (eGFR, CKD, and BUN) were analyzed as outcomes. Genetic variants associated with SRT were selected as instrumental variables using a significance threshold of *P* < 5 × 10^−6^ due to the limited availability of genome-wide significant variants. Although this relaxed threshold may increase the risk of weak-instrument bias, all included SRT instruments had F-statistics > 10, indicating adequate instrument strength. Linkage disequilibrium clumping was performed using r^2^ < 0.001 within a 10,000 kb window. The subsequent harmonization procedures, MR methods, and sensitivity analyses were conducted following the same approach as described above. The detailed F-statistics for each SNP are provided in Supplementary Tables 4–5.

## 3. Results

In this MR, we examined whether genetically predicted kidney-related traits, namely eGFR, CKD, and BUN, are causally associated with SRT. Robust genetic instruments were selected for each exposure, with all F-statistics exceeding 10, indicating adequate instrument strength. Multiple MR approaches, including both global and non-global weighting methods, were applied to strengthen causal inference.

**Figure 2.**
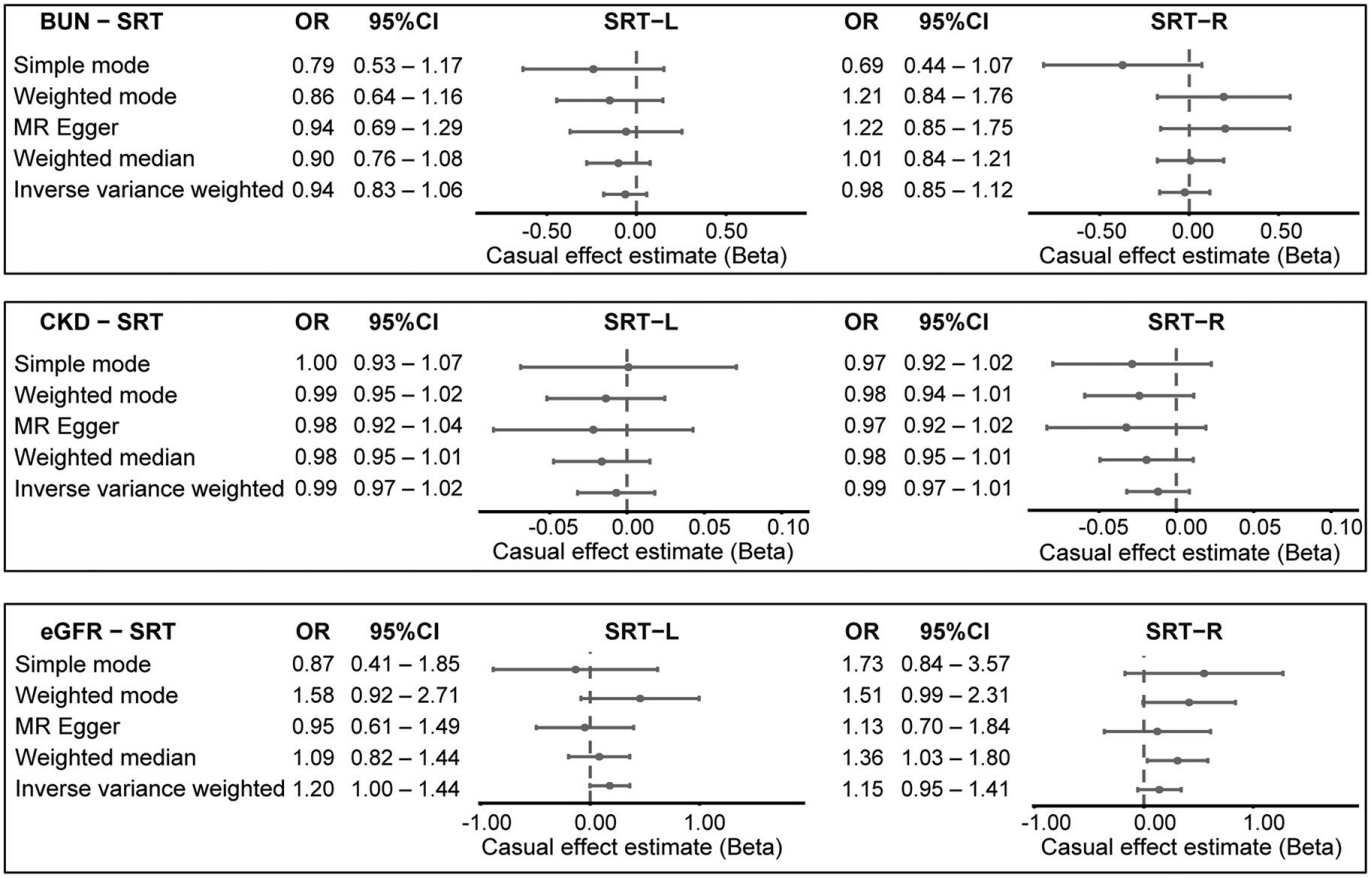
Forest plot of Mendelian randomization analyses showing the causal relationships of BUN, CKD, and eGFR with SRT, presented as effect estimates and 95% confidence intervals.

Across multiple MR methods, genetically predicted BUN, CKD, and eGFR showed no statistically significant causal associations with speech reception threshold in either ear (Table 1 and Figure 2). Specifically, genetically predicted BUN was not associated with SRT in the left ear (IVW: *β* = −0.0625, SE = 0.0615, *P* = 0.309) or the right ear (IVW: *β* = −0.0253, SE = 0.0717, *P* = 0.724). Similarly, genetically predicted CKD showed no significant association with SRT in the left (IVW: *β* = −0.0070, SE = 0.0127, *P* = 0.585) or right ear (IVW: *β* = −0.0118, SE = 0.0103, *P* = 0.252). Consistently, no significant causal effect of eGFR on SRT was observed for either the left ear (IVW: *β* = 0.1803, SE = 0.0932, *P* = 0.0529) or the right ear (IVW: *β* = 0.1422, SE = 0.1014, *P* = 0.1608). The statistical power analysis indicated that the study was able to detect minimum causal effect sizes of approximately *β* = 0.04–0.06 for eGFR, *β* = 0.07–0.09 for BUN, and *β* = 0.12–0.16 for CKD at 80% power and a significance level of 0.05. These effect sizes are well within the range of effects reported in observational studies, reinforcing that the null finding is not due to inadequate power.

**Table 1.** The results of IVs used for analyzing the effects of BUN, CKD, and eGFR on SRT.

Exposure	Outcome	Method	nSNP	Beta	SE	*p*-value
BUN	SRT-L	Inverse variance weighted	68	−0.063	0.061	0.309
		MR Egger	68	−0.058	0.160	0.718
		Weighted median	68	−0.101	0.090	0.263
		Simple mode	68	−0.240	0.201	0.236
		Weighted mode	68	−0.149	0.151	0.327
BUN	SRT-R	Inverse variance weighted	68	−0.025	0.072	0.724
		MR Egger	68	0.201	0.184	0.279
		Weighted median	68	0.007	0.095	0.938
		Simple mode	68	−0.373	0.226	0.104
		Weighted mode	68	0.192	0.189	0.313
CKD	SRT-L	Inverse variance weighted	22	−0.007	0.013	0.585
		MR Egger	22	−0.022	0.033	0.515
		Weighted median	22	−0.016	0.016	0.306
		Simple mode	22	0.001	0.036	0.977
		Weighted mode	22	−0.014	0.019	0.489
CKD	SRT-R	Inverse variance weighted	22	−0.012	0.010	0.252
		MR Egger	22	−0.032	0.026	0.232
		Weighted median	22	−0.019	0.015	0.211
		Simple mode	22	−0.029	0.026	0.286
		Weighted mode	22	−0.024	0.018	0.197
eGFR	SRT-L	Inverse variance weighted	194	0.180	0.093	0.053
		MR Egger	194	−0.047	0.227	0.835
		Weighted median	194	0.083	0.143	0.561
		Simple mode	194	−0.134	0.384	0.727
		Weighted mode	194	0.457	0.276	0.099
eGFR	SRT-R	Inverse variance weighted	191	0.142	0.101	0.161
		MR Egger	191	0.124	0.248	0.616
		Weighted median	191	0.308	0.142	0.030
		Simple mode	191	0.549	0.368	0.137
		Weighted mode	191	0.414	0.216	0.057

Sensitivity analyses supported the robustness of the MR estimates for the associations of BUN, CKD, and eGFR with SRT (Table 2). MR-Egger intercept tests showed no evidence of horizontal pleiotropy for any of the exposures in either ear. Cochran’s Q tests indicated no heterogeneity for most exposure–outcome pairs, although mild heterogeneity was observed for BUN with right-ear SRT and for eGFR with right-ear SRT. MR-PRESSO analyses did not detect significant distortion in the causal estimates, and outlier-corrected results were highly consistent with the primary estimates, indicating that the findings were not materially influenced by pleiotropic or outlying variants.

**Table 2.** Sensitivity analysis of the results of MR analysis of BUN, CKD, and eGFR on SRT.

Exposure	Outcome	*p* for pleiotropy^a^	*p* for Cochrane’s Q^b^	MR-PRESSO (Raw)			MR-PRESSO (Outlier-corrected)				*p* for global test	*p* for distortion test^c^
				*Β*	Sd	*p*	*β*	Sd		*p*		
BUN	SRT-L	0.975	0.355	−0.063	0.061	0.313	NA	NA		NA	0.370	NA
BUN	SRT-R	0.187	0.011	−0.025	0.072	0.725	−0.054	0.067		0.421	0.011	0.785
CKD	SRT-L	0.629	0.061	−0.007	0.013	0.590	NA	NA		NA	0.072	NA
CKD	SRT-R	0.405	0.741	−0.012	0.009	0.210	NA	NA		NA	0.757	NA
eGFR	SRT-L	0.273	0.092	0.180	0.093	0.054	NA	NA		NA	0.093	NA
eGFR	SRT-R	0.937	0.001	0.142	0.101	0.162	NA	NA	NA		0.002	NA

^a^*P*-values for pleiotropy were derived from MR-Egger test and *P*-value < 0.05 indicates a possible pleiotropic effect.

^b^Heterogeneity was assessed using Cochran’s Q statistic under the IVW model, and the corresponding *P*-value is reported.

^c^*P*-values for distortion were derived from MR-PRESSO test and *P*-value < 0.05 indicates a difference between estimates before and after outlier removal.

**Figure 3.**
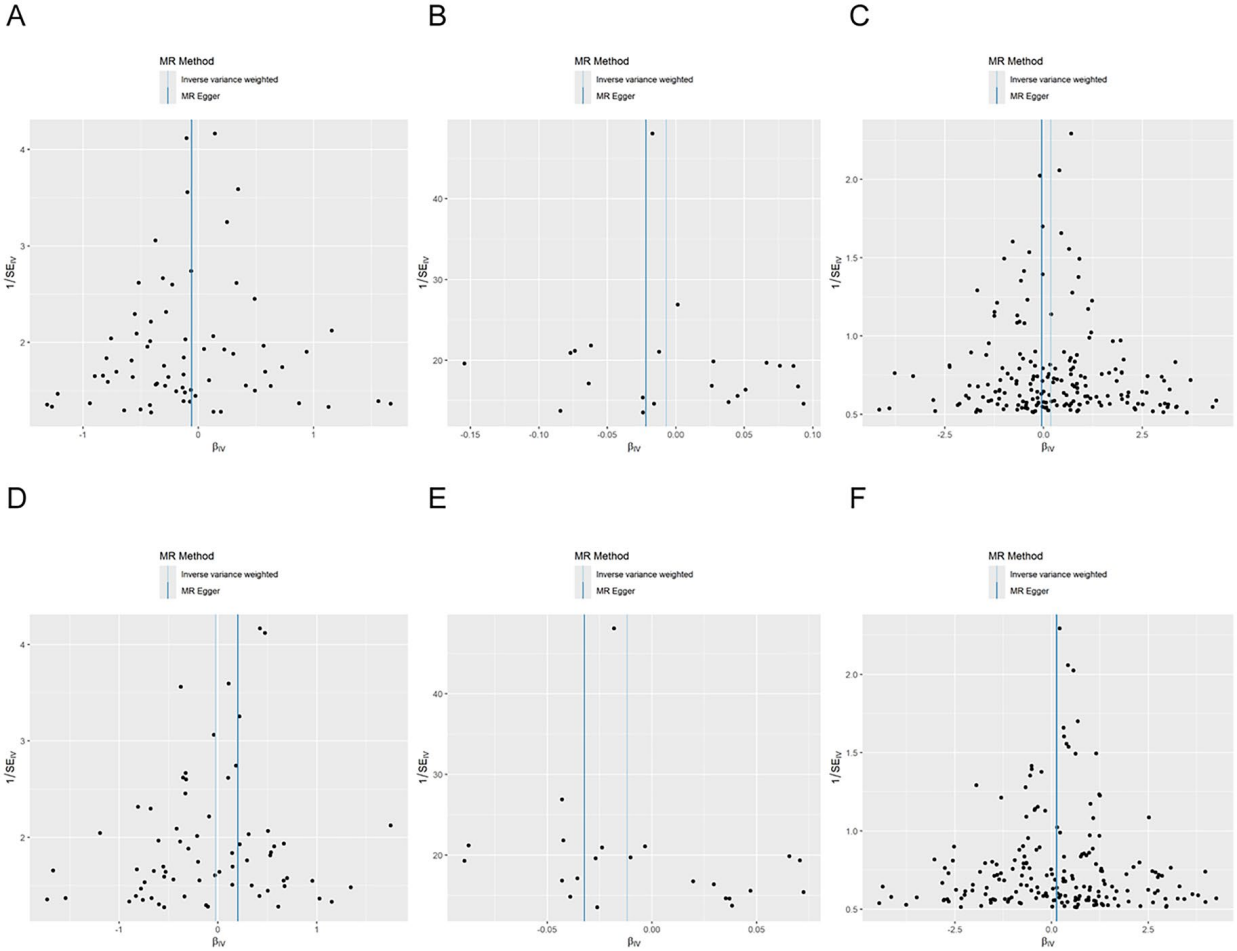
Funnel plots assessing heterogeneity in MR analyses of BUN, CKD, and eGFR on SRT. A–C show the funnel plots for left-ear SRT, corresponding to genetically predicted BUN, CKD, and eGFR, respectively. D–F show the funnel plots for right-ear SRT. Each point represents an individual SNP, and the symmetry of the points around the vertical line indicates the absence of directional pleiotropy.

Funnel plots demonstrated a largely symmetrical distribution of SNP-specific estimates around the IVW effect (Figure 3) and leave-one-out analyses confirmed that no single SNP disproportionately influenced the overall results (Supplementary Figure 1). The distributions of SNP-level associations and regression slopes across different MR methods are presented in Supplementary Figure 2. Overall, these results support the conclusion that there is no significant causal effect of kidney function traits on auditory function as measured by SRT.

To explore potential reverse causality, we performed bidirectional MR analyses using SRT (left and right) as the exposure and kidney function–related traits (eGFR, CKD, and BUN) as outcomes. Across all methods and both ears, no significant causal effects were observed (all *P* > 0.05). The full MR results are summarized in Supplementary Table 6 and Supplementary Figure 3.

## 4. Discussion

To our knowledge, this study is the first Mendelian randomization analysis specifically designed to evaluate whether genetically predicted kidney function traits influence speech reception threshold. Using multiple complementary MR approaches, we found no robust evidence supporting a direct causal effect of genetically predicted BUN, CKD, or eGFR on SRT in either ear. Although the IVW estimate for eGFR and left-ear SRT approached nominal significance and isolated nominal associations were observed in selected secondary methods, these findings were not consistent across analyses and did not satisfy our prespecified criteria for robust causal inference. Overall, the results support the interpretation that the epidemiological association between kidney dysfunction and impaired hearing-related performance is unlikely to be explained by a strong direct causal pathway from kidney function traits to SRT.

Importantly, these findings should not be interpreted as contradicting the well-established biological and developmental connections between the kidney and the inner ear. Previous studies have shown that these organs share developmental pathways, structural features, and molecular mechanisms, including ciliary function, fluid transport regulation, and basement membrane composition [18,33]. Genes implicated in oto-renal syndromes, such as EYA1, SIX1, and PAX2, further illustrate that disturbances in shared developmental programs can affect both renal and auditory structures [34–36]. In this context, our null MR findings are more appropriately interpreted as arguing against a simple vertical causal chain whereby genetically predicted reductions in kidney function directly lead to impaired speech reception, rather than against shared kidney, ear biology per se. A more plausible interpretation is that the observed epidemiological association may reflect shared developmental architecture, shared systemic pathophysiology, or horizontal pleiotropy, in which common biological mechanisms influence both organs independently [37,38]. This conceptual distinction is important for avoiding overinterpretation of either the developmental literature or the present MR findings.

A major consideration in interpreting our results is the choice of auditory phenotype. SRT is a functionally relevant measure of hearing performance and is particularly attractive for large-scale genetic analyses because it is continuous, objectively derived, and available in large biobank resources. However, SRT reflects not only peripheral auditory sensitivity but also aspects of central auditory processing and cognition. This is especially relevant because CKD-related hearing impairment has often been described as predominantly high-frequency sensorineural hearing loss, likely involving peripheral cochlear structures such as the stria vascularis [39]. Since SRT primarily reflects speech-frequency performance, it may be relatively insensitive to isolated high-frequency deficits [40]. Accordingly, our findings should be interpreted specifically as showing no robust causal effect of kidney function traits on speech reception threshold, rather than excluding causal effects on all hearing phenotypes. Future MR studies incorporating frequency-specific pure-tone thresholds or other phenotypes more directly reflecting peripheral cochlear dysfunction are necessary to more definitively address this question.

Our sensitivity analyses generally supported the robustness of the primary findings. MR-Egger intercept tests did not indicate clear directional horizontal pleiotropy, and MR-PRESSO-corrected estimates were largely consistent with the primary analyses. Nevertheless, heterogeneity was observed in selected exposure–outcome pairs, particularly BUN with right-ear SRT and eGFR with right-ear SRT. Such heterogeneity may reflect biological diversity of the selected instruments, phenotype complexity, non-directional pleiotropy, or analytic differences across SNPs rather than a single uniform mechanism. Therefore, the null findings for these specific pathways should be interpreted cautiously. Consequently, the null findings for right-ear SRT should be interpreted with greater caution than those for left-ear SRT, and the absence of heterogeneity for left-ear analyses provides stronger support for the null in that ear. At the same time, because the heterogeneity was not accompanied by consistent evidence of directional pleiotropy or material distortion after outlier correction, it does not provide strong support for an alternative causal interpretation.

Another important point is that the exposure definitions used here may not capture all clinically relevant aspects of kidney dysfunction. CKD was modeled as a binary trait using a conventional threshold, which is clinically practical but may obscure non-linear effects or associations confined to more advanced disease stages. It is possible that auditory consequences, if present, emerge predominantly in severe CKD, dialysis-dependent disease, or subgroups with particularly high vascular, metabolic, or inflammatory burden, as recently demonstrated in a Turkish cohort of patients with CKD and systemic inflammatory disease [41]. Likewise, MR based on lifelong genetic predisposition may be less sensitive to short-term physiological interactions or acute pathological changes that affect both kidney and auditory function in clinical settings. For these reasons, the present null findings should not be interpreted as excluding all biologically meaningful kidney–hearing relationships.

We also acknowledge several methodological limitations. First, complete exclusion of sample overlap between large consortia-based GWAS resources cannot be guaranteed. Although strong instrument strength reduces the risk of major weak-instrument bias, overlap remains a recognized limitation in two-sample MR and may affect precision or interpretation, particularly for near-threshold results [42]. Sample overlap, if present, would bias MR estimates toward the observational association; our null findings suggest this bias is not large enough to create a false positive, but it could contribute to type II error. Second, although we assessed pleiotropy and heterogeneity using multiple statistical approaches, the independence assumption of MR cannot be empirically verified with certainty using summary-level data alone. We note that while horizontal pleiotropy is a plausible conceptual explanation for our null findings, the MR-Egger intercept tests did not provide direct empirical evidence for its presence; thus, this interpretation remains inferential rather than demonstrative. Future work incorporating multivariable MR, frequency-specific hearing phenotypes, and more granular renal phenotypes may provide a more complete understanding of the kidney–hearing relationship.

From a clinical perspective, our findings support the view that hearing impairment observed in CKD populations may be better understood as a marker of shared systemic disease burden rather than a simple direct consequence of reduced renal function alone. Aging, hypertension, diabetes, vascular injury, metabolic dysfunction, and inflammatory burden are all plausible contributors to the observed co-occurrence of renal dysfunction and hearing impairment. This interpretation does not diminish the clinical importance of hearing evaluation in patients with CKD; rather, it suggests that hearing abnormalities in these patients may reflect broader multisystem vulnerability and should be assessed within that context.

In this two-sample Mendelian randomization study, we found no robust evidence supporting a direct causal effect of genetically predicted eGFR, CKD, or BUN on speech reception threshold. These findings suggest that the previously reported epidemiological association between kidney dysfunction and hearing impairment may be driven more by shared systemic, developmental, or environmental mechanisms than by a simple direct causal pathway from renal dysfunction to impaired speech reception. However, because SRT does not capture all aspects of auditory dysfunction, especially high-frequency peripheral hearing loss, our results should not be interpreted as excluding causal relationships with other hearing phenotypes. Future studies are encouraged to adopt comprehensive auditory assessments including high-frequency pure-tone average (4–8 kHz), distortion product otoacoustic emissions (DPOAEs), and word recognition scores, together with more refined renal phenotypes, multivariable MR, and bidirectional designs, to clarify the complex interplay between kidney and auditory health.

## Supplementary Material

Supplemental Material

Supplemental Material

Supplemental Material

Supplemental Material

Supplemental Material

Supplemental Material

Supplemental Material

Supplemental Material

Supplemental Material

Supplemental Material

## Data Availability

The GWAS summary statistics for kidney function traits were obtained from the CKDGen Consortium (https://ckdgen.imbi.uni-freiburg.de/). Summary statistics for speech reception threshold were accessed *via* the IEU OpenGWAS platform (https://gwas.mrcieu.ac.uk/), derived from the UK Biobank resource. All datasets used in this study are publicly available. Additional data have been deposited in the Figshare repository and can be accessed *via* the permanent DOI: https://doi.org/10.6084/m9.figshare.31476949.
