# Supplementary material for: Appraising the causal relationship between kidney function and hearing performance: a two-sample Mendelian randomization study
Source: Ren Fail. 2026 May 14;48(1):2663653. doi: 10.1080/0886022X.2026.2663653 (PMC13178041; doi:10.1080/0886022X.2026.2663653)
Supplement: Supplemental Material [file IRNF_A_2663653_SM2257.docx]

**Supplementary Material**

The supplementary materials for this study include additional tables and figures supporting the Mendelian randomization analyses.

Supplementary Table 1. SNPs associated with genetically predicted blood urea nitrogen (BUN) used as instrumental variables in the Mendelian randomization analysis.

Supplementary Table 2. SNPs associated with genetically predicted chronic kidney disease (CKD) used as instrumental variables.

Supplementary Table 3. SNPs associated with genetically predicted estimated glomerular filtration rate (eGFR) used as instrumental variables.

Supplementary Table 4. SNPs associated with genetically predicted SRT (left) used as instrumental variables.

Supplementary Table 5. SNPs associated with genetically predicted SRT (right) used as instrumental variables.

Supplementary Table 6. Results of bidirectional Mendelian randomization analyses.

Supplementary Figure 1. Leave-one-out (L00) plots displaying the influence of individual SNPs on the estimated causal effect. Panels A-C show the results for genetically predicted BUN, CKD, and eGFR on left-ear SRT, respectively, while panels D-F show the corresponding results for right-ear SRT.

Supplementary Figure 2. Scatter plots showing the associations between SNP effects on kidney function traits and SNP effects on SRT. A-C represent the associations for genetically predicted BUN, CKD and eGFR with left-ear SRT, respectively. D-F represent the corresponding associations with right-ear SRT. Each point represents an individual SNP, with the x-axis indicating the SNP-exposure effect and the y-axis indicating the SNP-outcome effect. Solid lines denote causal effect estimates derived from different Mendelian randomization methods.

Supplementary Figure 3. Bidirectional MR scatter plots. Panels A-C show the associations of genetically predicted left-ear SRT with BUN, CKD, and eGFR, respectively, while panels D-F show the corresponding associations for right-ear SRT.

All supplementary datasets supporting the findings of this study have been deposited in the Figshare repository and are publicly available at: <https://doi.org/10.6084/m9.figshare.31476949>.
