# Supplementary figures and images for: Appraising the causal relationship between kidney function and hearing performance: a two-sample Mendelian randomization study
Source: Ren Fail. 2026 May 14;48(1):2663653. doi: 10.1080/0886022X.2026.2663653 (PMC13178041; doi:10.1080/0886022X.2026.2663653)

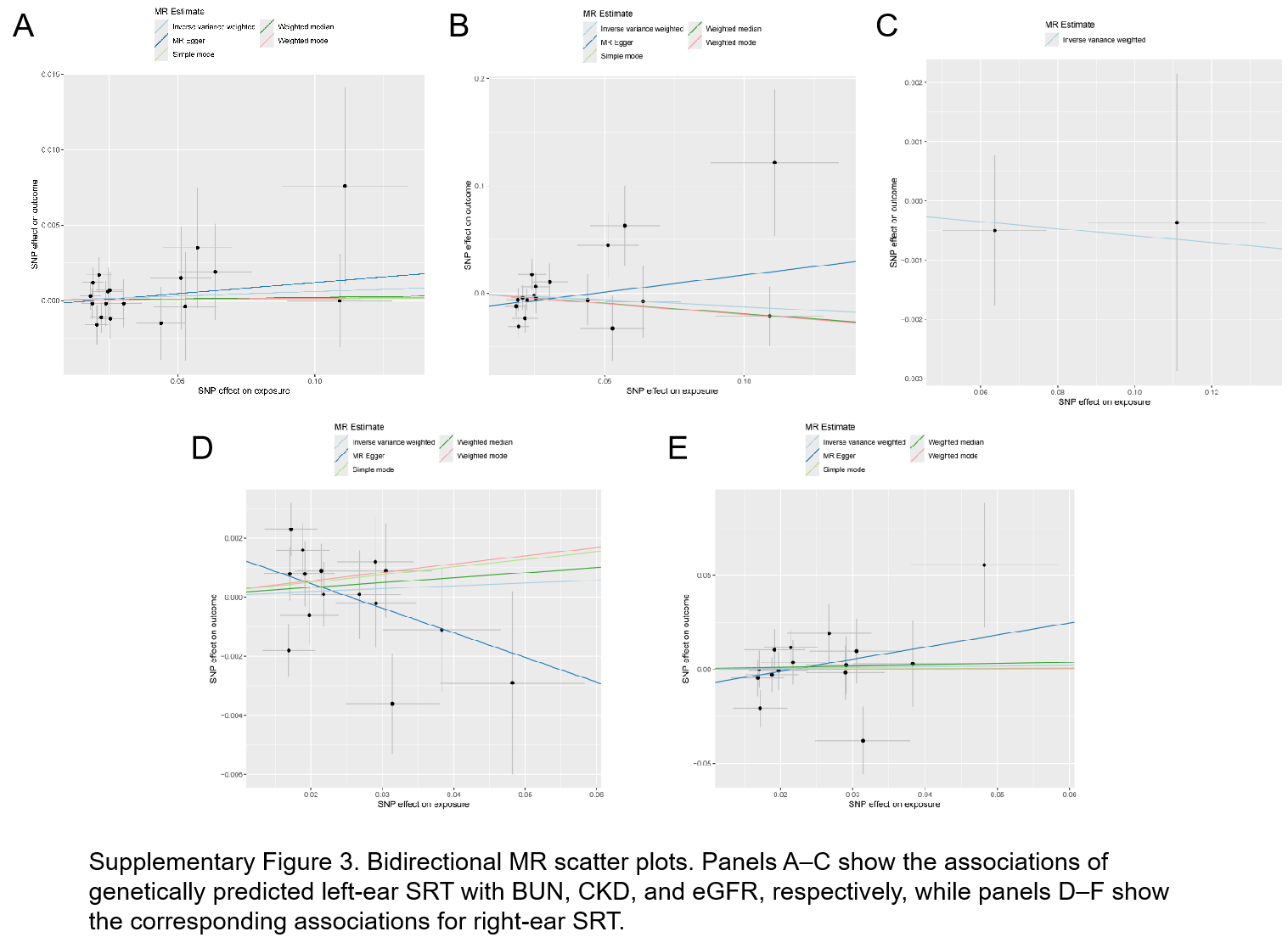

Supplement: Supplemental Material [file IRNF_A_2663653_SM2256.tif]

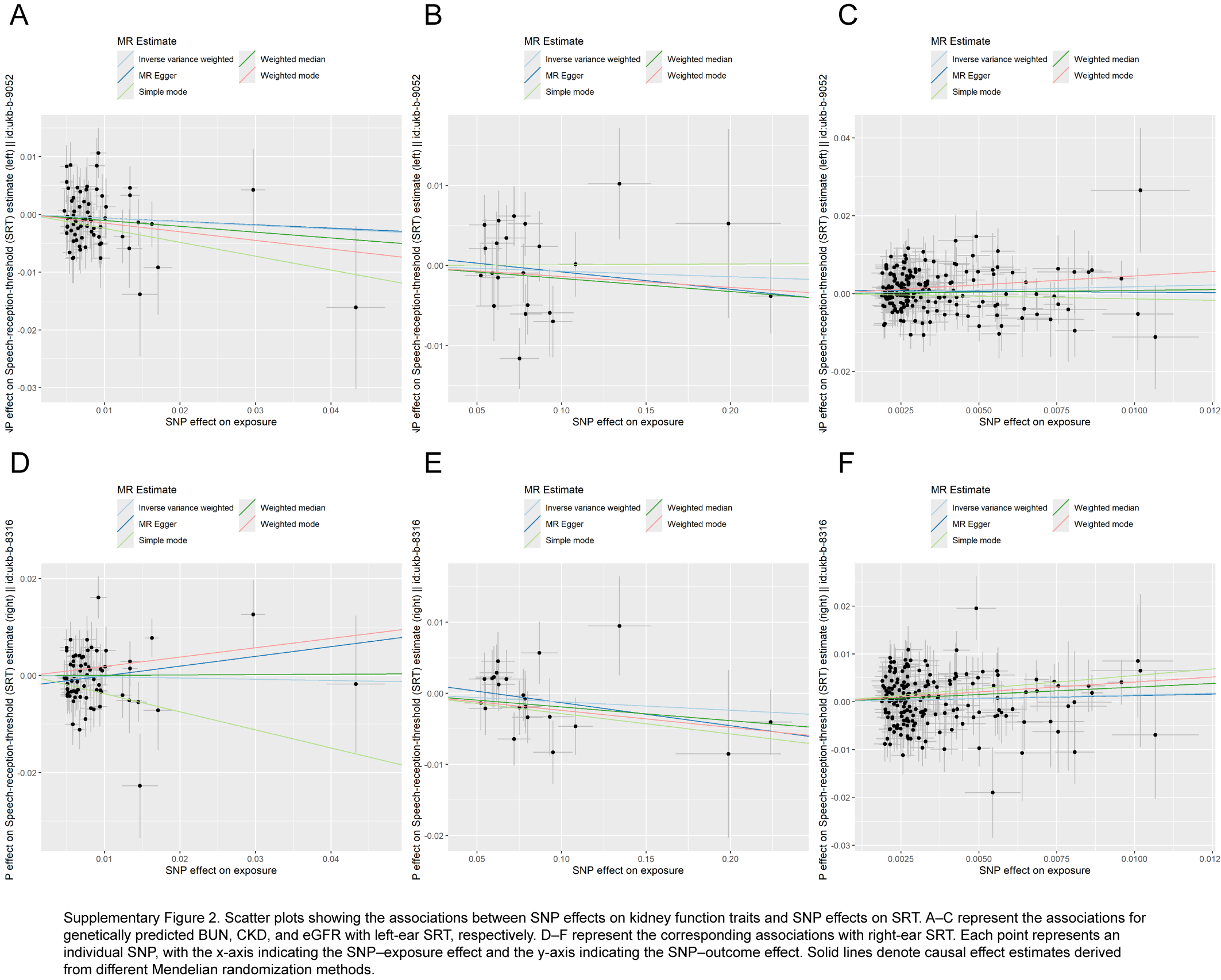

Supplement: Supplemental Material [file IRNF_A_2663653_SM2255.tif]

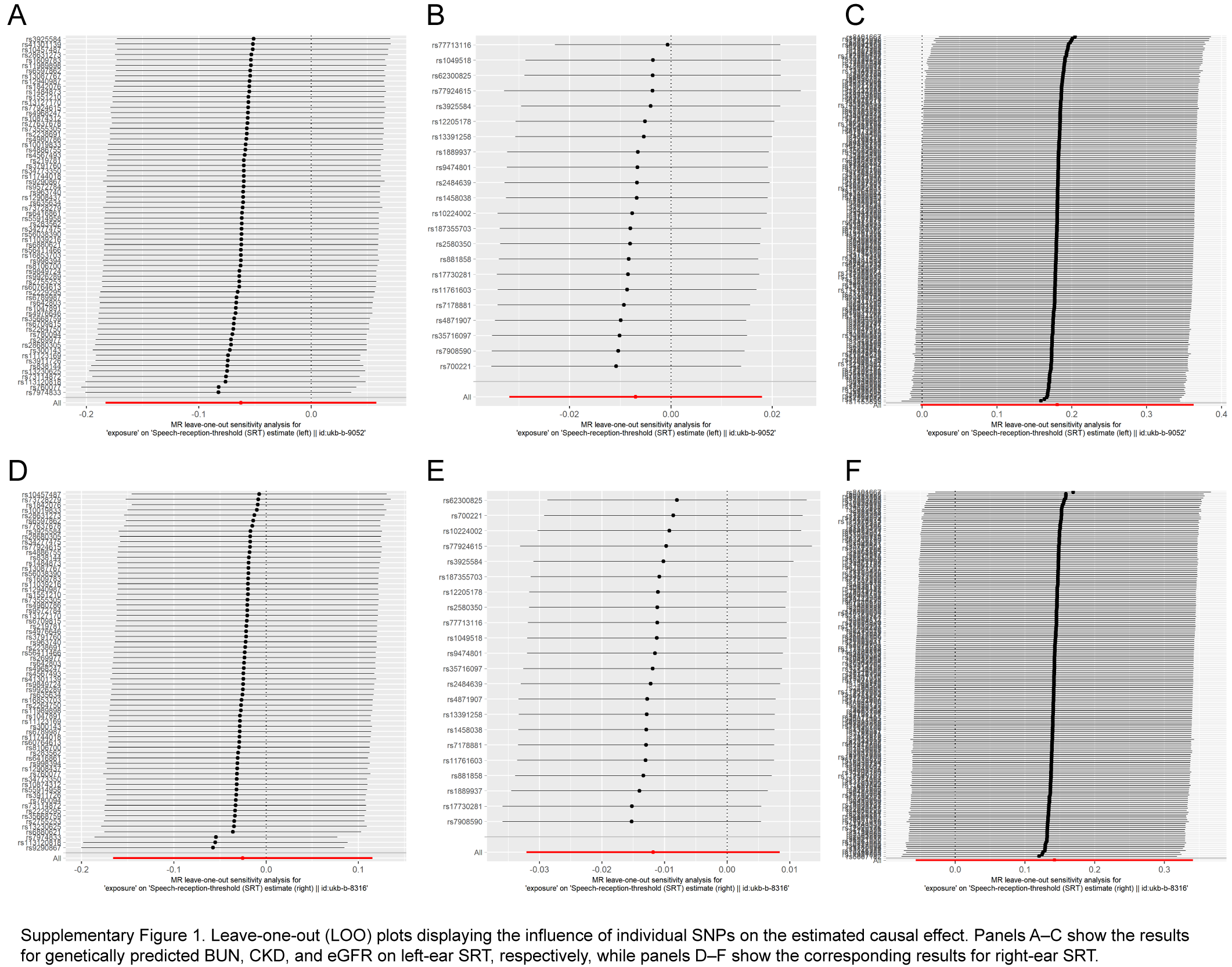

Supplement: Supplemental Material [file IRNF_A_2663653_SM2252.tif]
